# Toolkit to prompt and support stopping antidepressants in general practice: an interview study exploring patient participants’ experiences in the RELEASE trial

**DOI:** 10.1136/bmjopen-2026-116702

**Published:** 2026-05-21

**Authors:** Polyana Moura Ferreira, Suzanne McDonald, Katharine A Wallis, Maria Donald

**Affiliations:** 1 The University of Queensland, Brisbane, Queensland, Australia

**Keywords:** General Practice, QUALITATIVE RESEARCH, Health Services

## Abstract

**Abstract:**

**Objective:**

To explore patient experiences with the RELEASE (Redressing long-term antidepressant use) toolkit resources, including antidepressant hyperbolic tapering plans designed to minimise withdrawal symptoms as part of the RELEASE trial’s implementation evaluation to understand what aspects of the toolkit work well and identify areas where refinement could enhance future implementation. The RELEASE intervention, a multistrategy intervention codesigned with general practitioners (GPs) and patients to prompt and support stopping long-term (>12 months) antidepressants, is being evaluated in an effectiveness-implementation hybrid type-1 cluster randomised controlled trial in general practice.

**Study design:**

One-to-one semistructured qualitative interviews using reflexive thematic analysis to identify patterns of shared meaning.

**Setting:**

General practice, south-east Queensland, Australia.

**Participants:**

Adult participants (aged 18 years or older) taking an antidepressant long-term allocated to the intervention arm of the RELEASE trial.

**Results:**

31 participants with an average duration of antidepressant use of 16.5 years (range 1–30 years) were interviewed. Seven themes were identified. Across seven themes, participants described how the RELEASE toolkit resources prompted reflection on their antidepressant use, supported informed conversations with GPs, and offered tapering plans that were both structured enough to instil confidence and flexible enough to help participants feel in control of their tapering journey. They also identified barriers, including beliefs that very small tapering doses were unnecessary, difficulty accessing the mini-doses required for hyperbolic tapering and the challenge of managing withdrawal symptoms. For those who successfully stopped, the transition was associated with a renewed sense of autonomy. Participants also offered practical suggestions to strengthen the toolkit and its broader implementation.

**Conclusions:**

The RELEASE toolkit resources played a meaningful role in prompting and supporting informed shared decision-making and guiding attempts to stop antidepressants. Clearer messaging about the potential for withdrawal symptoms and access to mini-doses for tapering are needed to support patients to safely stop antidepressants.

**Trial registration number:**

ACTRN12622001379707p; Pre-results.

**STRENGTHS AND LIMITATIONS OF THIS STUDY**

- A strength of this study was the recruitment of a sample of participants varying in age, gender, antidepressant type and duration of use.
- Another strength is the inclusion of people at various stages of the tapering journey, from contemplation to successful completion.
- Interview participants were recruited from within a trial setting so findings may not be representative of tapering experiences outside this setting.
- People who agreed to be interviewed may hold stronger interest in engaging with the Redressing long-term antidepressant use toolkit resources than people who chose not to participate, meaning some perspectives may not be represented.

## Introduction

 Many people take antidepressants for longer than clinical guidelines generally recommend.^1 2^ Most clinical guidelines recommend antidepressant use to be temporary and emphasise the importance of appropriate treatment duration, typically recommending 6 to 12 months of antidepressant therapy for a single episode of moderate to severe depression.^3^,^6^ Yet in Australia, the average duration of antidepressant treatment is approximately 4 years.^7 8^ People continuing to take antidepressants long-term (>12 months) is contributing^12 9^,^11^ to the rising prevalence of antidepressant use observed globally, with estimates suggesting use has doubled in developed countries over the past 10 years.^12^

Studies indicate that patients often do not discontinue because they lack awareness of the need to stop antidepressants or incorrectly believe that treatment is intended to be lifelong.^2 13^ Moreover, many people experience withdrawal symptoms when trying to stop antidepressants, which are often not recognised and mistaken for a relapse or a continued need for medication.^14^ In qualitative research, fear of withdrawal symptoms is frequently cited as a key reason why people are hesitant to attempt stopping their antidepressant medication^2 15^ or a barrier to successful discontinuation.^16 17^ Additional factors that may contribute to rising long-term antidepressant use include lack of proactive medication review, financial barriers including consultation and medication costs, and prescriber fear about reducing antidepressants.^15 18 19^

Several strategies to support antidepressant discontinuation have been investigated for example primary care clinician prompted review,^20 21^ cognitive behavioural therapy,^22^ mindfulness-based cognitive therapy^23^ and internet and telephone support.^24^ Also, recent guidelines recommend hyperbolic tapering,^25^ where dose reductions are made in progressively smaller amounts and to very low doses (eg, sertraline 100 mg, 50, 25, 12.5, 9, 6, 4, 2, 1 mg), which can help to minimise withdrawal symptoms to enable people to stop.^1726^,^29^ However, implementing tapering in clinical practice remains challenging.^14 30^ Most antidepressant prescribing occurs within primary care settings, where the adoption of tapering plans is minimal and there is limited awareness and recognition of withdrawal symptoms.^3 4 31^ A further challenge is that most antidepressant medications are not available in the mini-doses required for hyperbolic tapering, and current clinical guidelines typically lack practical advice on tapering.^4 14 26 32 33^

Hyperbolic tapering of antidepressants is a core component of the RELEASE (Redressing long-term antidepressant use) intervention, which aims to prompt medication review and support informed shared decision-making about continuing or stopping antidepressant therapy.^34^ The RELEASE intervention is being tested in an effectiveness-implementation hybrid type-1 cluster randomised controlled trial in general practice. RELEASE targets both general practitioners (GPs) and patients taking antidepressants long-term. In both arms decisions about continuing or stopping antidepressants were made as usual by patients with their GP.

General practices in the intervention arm of the trial invited patients to attend a medication review. Intervention patients also received a study pack containing four resources, previously optimised in a think-aloud study with people with lived experience of long-term antidepressant use.^35^ The first was a brochure, *Stopping Antidepressants*, which raised awareness about long-term use, guideline recommendations, reasons people remain on antidepressants, and how to minimise withdrawal symptoms using hyperbolic tapering. A second brochure for family and friends offered advice on supporting someone stopping antidepressants. The third resource was a decision aid to help participants make an informed choice about continuing or stopping treatment, including a comparison of key pros and cons (eg, “I want to feel emotion again, highs and lows” vs “I would rather not feel the ups and downs”). The fourth resource was a structured, drug-specific tapering plan with step-by-step instructions for how to hyperbolically reduce antidepressant dose. The plans are flexible, enabling participants to slow down or return to a previous dose if needed. The plans also explain how to access the antidepressant mini-doses required for tapering. Initially the dose reductions may be achieved by cutting tablets, but at smaller doses compounded mini-dose capsules or liquids available via compounding pharmacies are usually required, or patients may access off-label mini-doses by crushing tablets and mixing with water, or by opening capsules to count or weigh individual beads.

This qualitative study formed part of the RELEASE trial’s hybrid type-1 implementation evaluation. The aim was to explore trial patient participants’ experiences regarding the RELEASE toolkit resources to inform and strengthen efforts for wider implementation.

## Methods

### Study design

A qualitative evaluation study interviewing patient participants allocated to the intervention arm of the RELEASE trial. The study is reported according to the Consolidated Criteria for Reporting Qualitative Research reporting guidelines.

### Participant recruitment

A purposive sample of participants from the RELEASE intervention arm was recruited to reflect the distribution of trial participants across age and gender. Potential participants were sent an email containing the Participant Information and Consent Statement, along with an invitation via email and/or SMS to take part in a telephone interview about the RELEASE resources. Up to two reminders were sent. Participants who confirmed their interest were then scheduled for an interview at a convenient time. All participants provided informed written consent before the interview. Sample size was context-dependent rather than predetermined;^36^ we used the concept of information power considering the quality of dialogue to ensure sufficient depth and relevance of the data.^37^

### Data collection

One-to-one semistructured telephone interviews were conducted with 31 participants. Interviews were conducted during the 12-month follow-up period of the trial. The interview guide was informed by the Consolidated Framework for Implementation Research interview guide tool (in particular the innovation-domain questions) and literature about stopping antidepressants.^15 38^ Using open-ended prompts, the interview guide focused on several key areas: participants’ perceptions of the RELEASE resources, in particular their drug-specific tapering plan; their decision-making about antidepressant discontinuation and whether or not they chose to taper; factors that supported successful discontinuation; reasons some participants continued their antidepressant despite exposure to the RELEASE intervention; and reflections on how the intervention could be improved. The interview guide was adapted during the data collection period to capture variability in tapering trajectories (eg, those who initiated but did not complete the tapering). Interviews were conducted by two female qualitative researchers trained in interviewing methods, who were not involved in delivering the intervention and had no prior relationship with participants. Interviews lasted between 15 and 40 min (average 23.5 min). All interviews were conducted via telephone, audio-recorded, transcribed verbatim, anonymised, and checked for accuracy by the first author.

### Data analysis

Data were analysed using reflexive thematic analysis, a method well suited to understanding experiences and meanings across qualitative data.^39^ Reflexive thematic analysis acknowledges the researcher’s subjectivity as a valuable resource, not a limitation.^39^,^41^ Our approach to reflexive thematic analysis was informed by a critical realist ontology, acknowledging that people’s experiences are shaped by real-world conditions such as their physical health, social contexts and institutions like the healthcare system. Relatedly, we draw from an interpretivist approach, focusing on how people who take antidepressants understand and use resources during their decision making and tapering journey. This combined lens enabled our team to explore both the broader influences and the personal narratives that shape how the RELEASE toolkit resources were experienced and interpreted.^42^

Reflexive thematic analysis was employed inductively, meaning that we stayed close to participants’ responses without relying on pre-existing frameworks. Both semantic codes (reflecting explicit content) and latent codes (capturing deeper underlying meanings and assumptions) were developed.^43^ The first author conducted initial familiarisation and coded all transcripts. Two additional researchers independently reviewed a subset of transcripts, contributing codes and annotations during their own familiarisation process. The research team engaged in a process of reflexivity through eight 90 min collaborative discussions to review, develop, and refine themes.^40^ We acknowledge that our analysis is shaped by our broader research interests and pursuits including general practice research, health services research, public health, psychology and implementation science. The team included a GP-clinician researcher. These perspectives informed the framing of our research aim, how interviews were conducted, and our interpretation of the findings and fostered a heightened sensitivity to issues such as the complexities surrounding how patients and GPs navigate antidepressant use and discontinuation, the general practice environment, and access to quality healthcare. Rigour was guided by Braun and Clarke’s guidance on conducting high-quality reflexive thematic analysis.^44 45^ Quirkos software supported data management throughout the analysis.^46^

## Results

31 semistructured interviews were completed. Table 1 illustrates the distribution of participant experiences in response to exposure to the RELEASE toolkit resources (ie, current tapering status) and describes participants’ characteristics, including baseline antidepressant use (duration and drug type) for each tapering status group. Participants had a mean age of 56 years (range 27–86 years) and included 8 men and 23 women. At the time of the interview, participants had been exposed to the RELEASE resources for a mean of 10.5 months (range 5–14 months), with those exposed for longer having had more time to reflect on the resources. The average duration of antidepressant use was 16.5 years (range 1–30 years). Nineteen participants had been taking an antidepressant for longer than 10 years. The most used antidepressant was sertraline, followed by escitalopram, fluoxetine, citalopram and venlafaxine. Desvenlafaxine, agomelatine and mirtazapine were used by only one or two participants. Exposure to the RELEASE resources prompted all 31 participants to reconsider their antidepressant use and 20 to begin decreasing their antidepressant dose of whom eight stopped taking antidepressants altogether. A key distinction is that these eight participants had taken antidepressants for a considerably shorter duration (5 years and 7 months on average) than, for example, those who did not complete their taper (19 years and 10 months). Eleven reported having a plan to decrease and stop at some stage in the future. A few participants had limited recall of the resources; this was more common among those who were still considering tapering or planning to do so in the future.

**Table 1:** Participant characteristics and antidepressant medication use at baseline by tapering status at the time of the interview

Tapering status	N	Gender	Mean age	Mean time between allocation and interview	Mean duration of use at baseline	Antidepressant medication type at baseline (n)
			Years	Months	Years and months (range)	
Stopped antidepressant	8	Male 4 Female 4	50	9	5 years 7 months (1–16 years)	escitalopram (3), citalopram (2), sertraline (2), fluoxetine (1)
Currently tapering antidepressant	4	Male 1 Female 3	61	10	16 years 4 months (9–21 years)	mirtazapine (1), venlafaxine (1), sertraline (1), desvenlafaxine (1)
Tapering initiated but not completed, with a reduced dose maintained	2	Male 0 Female 2	50	9	16 years 5 months (13–20 years)	citalopram (1), fluoxetine (1)
Initiated tapering but resumed antidepressant	6	Male 2 Female 4	63	11	19 years 10 months (10–30 years)	fluoxetine (2), citalopram (1), venlafaxine (1), desvenlafaxine (1), escitalopram (1)
Clear intention to taper in the future, with a planned start date	6	Male 1 Female 5	51	9	13 years 10 months (2.5–23 years)	sertraline (2), venlafaxine (1), fluoxetine (1), citalopram (1), agomelatine (1)
Contemplating tapering but no specific plan or time frame	5	Male 0 Female 5	44	11	15 years 6 months (3 –26 years)	sertraline (2), venlafaxine (1), duloxetine (1), escitalopram (1)
Total	31	Male 8 Female 23	56	10.5	16 years 7 months (12–22 years)	sertraline (7), escitalopram (5), fluoxetine (5), citalopram (5), venlafaxine (4), desvenlafaxine (2), agomelatine (1), duloxetine (1), mirtazapine (1)

Seven themes, one with two subthemes, were identified. Themes, subthemes and the central organising concepts for each are described in table 2. Supporting quotes indicate the participant’s age, gender, and antidepressant use duration.

**Table 2:** Themes, subthemes and central concepts

Theme/subtheme	Central concept
Theme 1: Prompting and enabling informed decision-making	Demonstrates how the RELEASE resources prompted participants to reconsider their antidepressant use, emboldened them to engage with their GP and enabled informed decision-making.
Theme 2: The importance of the GP in decision-making	Depicts how GPs can influence participants in either direction, to continue or attempt to taper and stop antidepressants.
Theme 3: Structured tapering plans are empowering	Describes how the tapering plans helped patients to visualise a way forward and gave them the confidence to try, while the flexibility enabled patients to remain in control of their own tapering journey.
Theme 4: The barriers to hyperbolic tapering	Describes why many participants did not follow the plans through the lower doses.
Subtheme. Perceived lack of need for hyperbolic tapering through to very low doses	Reflects the belief that hyperbolic tapering to such small doses was not necessary.
Subtheme. Poor access to antidepressant mini-doses needed for hyperbolic tapering	Accessing the requisite mini-doses was too difficult or too expensive to be worthwhile.
Theme 5: The power of withdrawal symptoms	Describes how participants gave up attempting to decrease their antidepressant dose, sometimes pressured by family, because the withdrawal symptoms were too awful and disruptive to their lives.
Theme 6: Reclaiming self: transitioning to an antidepressant free life	Captures how successfully stopping antidepressants fostered a sense of liberation and confidence.
Theme 7: Modifications to enhance broader implementation	Summarises participants’ suggestions for support and raising awareness about recommended duration of antidepressant therapy and how to safely stop antidepressants.

GP, general practitioner; RELEASE, Redressing long-term antidepressant use.

### Theme 1: prompting and enabling informed decision-making

This theme reflects how the RELEASE resources, including the two information brochures, decision aid and tapering plans, prompted participants to reconsider their relationship with antidepressants. For example, one participant had this to say about the information in the brochures:

I suppose that’s why I did try and wean myself off for a while and was very hesitant to go back on after reading the facts in the brochures. (4, F, 70+yrs, 10+yrs on antidepressants).

Discovering that antidepressants do not always need to be taken for life was a revelation for some participants, increasing their confidence to speak with their GP and explore options.

After reading in the brochure that you’re only supposed to be on it for six to 12 months or whatever. No doctor has ever told me that. So, that was an eye opener for me. (P24, M, 50-60yrs, <5yrs on antidepressants)

One participant said that the brochure helped to empower them to initiate a conversation with their GP about their mental health and antidepressant medication, and that the information in the brochure could speak for them, especially as they had felt dismissed in the past.

I am always really self-conscious and shy talking to GPs about my mental health it was easier to put the brochure in front of them. I literally just said, hey, I’m part of this thing. What do you think? So, having the brochure took the onus off me and put it on the doctor to continue the conversation. (P18, F, 40-45yrs, 5-10yrs on antidepressants)

The decision aid also played a role in validating participant decisions. For some, the decision aid was useful as it highlighted the benefits of stopping, while for others, it confirmed that their decision to keep taking antidepressants was the right decision for now.

The list [of pros and cons on the decision aid] said if you’re feeling this way, that way maybe it’s not a good time to stop. It gave me permission to go, okay, I still need this Even though part of me wants to quit. (P18, F, 40–50yrs, 5-10yrs on antidepressants)

Exposure to the RELEASE resources prompted a determination to stop antidepressants for some participants.

I just got on with it. I think I was just very pragmatic about it. I’m gonna go do this. I’m gonna commit to it and if I managed to get through the three months of crushing pills and having the little things, then great and if I don’t, then, okay, that’s also fine. (P11, M, 40-50yrs, 5-10yrs on antidepressants)

### Theme 2: the importance of the GP in decision-making

Some participants commented how their GP encouraged them to taper and provided guidance on the approach, which motivated them to attempt to stop antidepressants, while other participants said that their GP was ambivalent or non-committal or actively discouraging. Participants were more likely to attempt to stop antidepressants and follow a RELEASE tapering plan when their GP supported the approach, with the GP sometimes acting as the catalyst for a patient starting on this journey.

The fact that [GP] has put me up for it made me think that again, [GP] has always got us in mind and [GP] knows my motivations, and knows my situation really, really well. (P22, F, 50-60yrs, 10+yrs on antidepressants)

Some participants described their GP as non-committal, and this made them less inclined to initiate tapering. One felt that if their GP had encouraged them more, they might have been more willing to attempt stopping antidepressants.

I think [GP] could have gone in and sort of challenged me a little bit, but that didn’t happen. [GP] just sort of said, well, you are on a lower dose, and if it’s not something that you really want to do and go through, then keep doing what you’re doing. I think that if [GP] challenged me, it would have given me a bit more motivation to have a go. (P16, F, 60-70yrs, 10+yrs on antidepressants)

There was recognition that there is often not enough time to discuss tapering with the GP, and without this discussion tapering would not be started.

I thought about it and talked to the doctor about it, and it was always sort of like, yeah, well, we can discuss that next time, when we have more time within the appointment. But we never actually discussed it. And then we never actually got into the reduction. (P12, F, 40-50yrs, 10yrs on antidepressants, venlafaxine, contemplating tapering but no specific plan or timeframe)

One participant expressed frustration that their GP discouraged them from stopping antidepressants, even though they felt well and as if they no longer needed antidepressants. Nevertheless, they trusted the GP’s judgment and followed their advice to continue medication.

I felt I was dealing with the situation a lot better, and I wasn’t stressing out like I was before. That’s why I kind of said to [GP], “I’m feeling I’m better. I’d like to get off them as quick as possible” but [GP] was still not convinced and wanted me to stick with it. But, I don’t want to be on these things because I’d rather just deal with things as I can rather than have to need medication to do so. (P23, M, 60-70yrs, 2.5yrs on antidepressants, agomelatine, clear intention to taper in the future with a planned start date)

### Theme 3: structured tapering plans are empowering

Participants said the plans helped them to visualise a way forward and provided a way to track their progress. The RELEASE hyperbolic tapering plans gave participants the confidence and motivation to start tapering, providing clarity and a sense of being supported.

I took it every time to my appointment as well. Yes, definitely help… You can actually have the track of when you started and what you did, and how you did. (P9, F, 50-60yrs, 16yrs on antidepressants, fluoxetine, stopped antidepressant)

Some participants felt reassured that their tapering plan was based on credible, research-based information. Participants felt that they could trust the plans.

So, in a way, for me, it was kind of this is being done by a professional people that really looked into mental health, depression, anxiety, so that, I think that’s why I’ve been more confident. (P4, F, 70-80yrs, 30yrs on antidepressants, desvenlafaxine, initiated tapering but resumed antidepressant)

The tapering plans enabled people who had previously tried but not managed to stop antidepressants to see and try a different approach. By offering a more logical and manageable process than past attempts, the plan gave them the courage to try again.

I tried to come off it before, but I wasn’t told the information as being able to go down the way it was… I was told to take it every second day and then just come off it, and so when I tried before, it didn’t work very well, and so I thought this would be better. (P28, F, 50-60yrs, 5-10yrs on antidepressants)

Participants appreciated the flexibility of the RELEASE tapering plans, saying that this flexibility made the process feel more personalised, manageable, and empowering.

I feel pretty confident about being able to taper until I’m completely off Pristiq, and I get that confidence because of the number of steps to reduce the dose… you can extend, you can shorten there’s enough flexibility in there. (P5, F, 40-50yrs, 5-10yrs on antidepressants)

### Theme 4: barriers to hyperbolic tapering

Of the participants who attempted to decrease their antidepressant dose, few followed the RELEASE hyperbolic tapering plans through the lower doses to completion.

#### Perceived lack of need for hyperbolic tapering through to very low doses

Some participants said that the RELEASE hyperbolic tapering plans were too long or complicated and they did not follow the plans, but opted instead to take antidepressants on alternate days or cut tablets. One participant attempted to approximate dose reductions, accepting that these rough estimates were adequate.

I just decided to halve the dose and wait to see how it feels. If I was feeling good, then I’ll just do, like one every other day, then a half every other day. It was like I’m following how I was feeling. I didn’t follow your tapering protocol at all. I feel like your tapering protocol was a lot lengthier and longer than I needed. (P26, F, 40-50yrs, <5yr antidepressants)

While the RELEASE resources motivated people to stop antidepressants and to have a conversation with their GP many chose to stop more quickly than outlined in the tapering protocols or even to go “cold turkey.”

So obviously the literature and the conversation was the inspiration, the catalyst, but I just went cold turkey. When I make up my mind to do something, I just do it. (P25, F, 60-70yrs, <5yrs on antidepressants)

GP advice sometimes led participants to disregard the RELEASE tapering plans and instead follow their GP’s recommended approach to stopping antidepressants.

[The GP] said go and get yourself a pill cutter, cut them in quarters, do one week on three quarters, one week on a half, one week on a quarter, and taper from there. And just note how you feel. (P13, M, 60-70yrs, <5yr on antidepressants)

One participant chose to follow the RELEASE hyperbolic tapering plan rather than their GPs’ advice. This participant said that the RELEASE tapering plan guidance was more comprehensive and better suited to their needs.

Initially, my GP said…d take one [tablet] then miss a day, then miss two days. Anyway, I found the information in your review it covered more, and the GP steps were not the same I felt your tapering process… a better way of doing it, rather than the GP way of doing it. (P4, F, 70-80yrs, 10+yrs on antidepressants)

#### Poor access to antidepressant mini-doses needed for hyperbolic tapering

Hyperbolic tapering at low doses was often described as out of reach due to the high cost of compounded antidepressant mini-doses required for the latter stages of the hyperbolic tapering plan.

It [speaking about compounding] was going to cost me $85 [AUD]. So that’s a lot of money to me because I’m a single person and then my normal tablets are only $6.99 to $8.99. I did want to get off it, but I wasn’t willing to pay hundreds of dollars a month to get off it. All the paperwork is very good and it looks really simple to decrease but I just can’t afford it. (P15, F, 60-70yrs, 10+yrs on antidepressants)

Participants also identified challenges in finding a compounding pharmacy as another barrier to following a hyperbolic tapering plan.

The road block I had was actually finding a compounding pharmacy that understood what I was trying to do and I had a little bit of ohh, we don’t do it here, even though my GP sent me to that particular pharmacy. (P5, F, 40-50yrs, 5-10yrs on antidepressants)

The RELEASE tapering plans include, for some antidepressants, instructions for how to crush tablets and mix with water to make a dilute solution for mini-doses as a cheaper mini-dose alternative to compounded capsules. However, participants expressed concerns about the practicality preparing liquid solution daily (eg, while travelling) and commented that it felt too complex or time-consuming.

I did the compounded capsules because that’s just so much easier and I couldn’t be bothered doing it the other way. Too complicated. (P28, F, 50-60yrs, 5-10yrs on antidepressants)

Concerns were also raised about maintaining dose accuracy when using the liquid preparation.

I prefer capsules so I know what I’m taking, or I don’t have to try and judge what I’m supposed to be taking. (P15, F, 60-70yrs, 10+yrs on antidepressants)

However, two participants preferred making their own liquid preparation, saying it provided a more affordable and flexible option.

Just the convenience of just having it on you. I also had little measuring syringes, because I’ve got small kids So, I had everything I need to just go ahead and do that…Low effort, it was low cost, which was important. You’re just kind of in control; you can repeat a week if I need to. (P11, M, 40-50yrs, 5-10yrs on antidepressants)

### Theme 5: the power of withdrawal symptoms

The journey of decreasing and stopping antidepressants was rarely straightforward and seldom followed a linear process. While some participants began with clear intentions to decrease or stop antidepressants, their journey was often disrupted by withdrawal symptoms, which made it difficult for them to continue on the journey. The participants who successfully stopped antidepressants typically described only mild withdrawal symptoms such as ‘only some dizziness’ or ‘maybe a bit of nausea’, but those who paused or gave up tapering tended to report more severe withdrawal symptoms that caused substantial disruption to daily life.

It was probably about five weeks into it, and I found that I was very, very emotional and suffering a lot of anxiety and so I spoke to my doctor about it and they said that ‘at this stage, it’s not right for you. You can give it another couple of weeks and see how you go.’ And I thought, ‘Well, okay, if I do that, I know that the GP is there for me.’ It didn’t really improve at all; it just got worse. And I thought I really can’t cope with it anymore. (P27, F, 70-80yrs, 10+yrs on antidepressants)

Pressure from family or friends to stay on or restart antidepressant medication made stopping even more difficult for some participants.

Some of my family members are very strong on keeping up with medication for life, and don’t really understand why I would want to [referring about stop], and then obviously, if any little withdrawal symptoms, and then it’s like, you need to go back on, you need to increase. It’s a lot of pressure. (P8, F, 40-50yrs, 10+yrs on antidepressants)

Withdrawal symptoms left some participants feeling discouraged and unsure about whether to try again. Two participants ultimately resolved to remain on antidepressants.

So, I just went straight back on it and just accepted that it’s something that I need. (P1, M, 50-60yrs, 10+yrs on antidepressants)

Some participants who were not able to successfully stop despite trying, emphasised that tapering setbacks should not be seen as personal failure. They also highlighted their need for support when attempts to stop were unsuccessful.

And I think that’s my biggest message is more support and more information around [what to do if you’re feeling you can’t cope with tapering] and don’t feel bad if you stop but there is support around if you’re not successful. (P29, F, 70-80yrs, 10+yrs on antidepressants)

Participants reported fear of their ability to cope without medications and some reported fear of withdrawal symptoms, which meant they were hesitant to begin decreasing their antidepressant.

…the fear of the withdrawals, because I do suffer with them quite a bit when I get weaned off the medication. Especially trying different antidepressants which sort of pushed me again not to stop it. (P6, F, 20-30yrs, 1-5yrs on antidepressants)

### Theme 6: reclaiming self: transitioning to an antidepressant-free life

Eight of the 31 participants had successfully stopped antidepressants at the time of interview. Having stopped, they said how they valued reconnecting with their emotions, regaining a sense of control and feeling more like themselves.

When you’re on them, you can get very level-headed, and you can really control any emotion, which is great, that’s what they’re for. But it also means you’re not actually dealing with any emotions in life. So actually, having that back in my life is like, I’m a single dad with a daughter I want her to learn about emotions. (P2, M, 40-50yrs, 5-10yrs on antidepressants)

Some described feeling liberated and a renewed sense of empowerment in discovering that they could cope without medication.

And I do remember that when I stopped I was just happy that I was off them and it was awesome to see that I can actually function exactly the same without the medication. (P9, F, 50-60yrs, 10+yrs on antidepressants)

### Theme 7: modifications to enhance broader implementation

This theme reflected participants’ practical suggestions for modifying the RELEASE intervention for broader implementation.

Participants suggested that a mobile phone application (app) that included the tapering plans and enabled individuals to track their tapering progress and severity of withdrawal symptoms would be helpful and would provide reassurance.

An application on your phone where you can track the symptoms that you have as you’re tapering down your medication everything in one little place. (P9, F, 50-60yrs, 10+yrs on antidepressants)

Easier, wider access to the tapering plans and other RELEASE toolkit resources via a website(s) was identified as an important future priority. Electronic access to the resources was considered important to prompt informed decision-making about stopping antidepressants. One participant highlighted the importance of having access to the supporting research evidence.

I’m the sort of person that likes information, so I’d be interested in knowing that research has been done on how to come off them. I’d like bodies of research that I could see through and look at on a website to make my decision. (P11, M, 40-50yrs, 5-10yrs on antidepressants)

Many participants suggested enhanced support during the tapering process, through either Facebook groups that could provide support and shared experiences, or a dedicated telephone support line.

I think if I was tapering and I’m having a really bad day that [a telephone support line] would be helpful. To be able to not give up like I did at the end [speaking about previous experience of tapering outside of the trial] And I feel sad about that. I gave in too early. (P16, F, 60-70yrs, 10+yrs on antidepressants)

Others advocated for access to face-to-face psychological support while tapering. However, financial barriers were a concern, prompting calls for government-funded initiatives to make such services affordable.

If people are going to be stopping their antidepressants… they need some other support mechanisms in place to at least help with the transition. So, I think the promotion of more, you know, subsidised or free psychology appointments or psychiatry appointments, whatever they need. (P12, F, 40-50yrs, 5-10yrs on antidepressants)

The importance of raising public awareness, often via social media channels, about the appropriate duration of antidepressant use, the effects of long-term antidepressant use, and antidepressant withdrawal symptoms were emphasised as a central focus for future efforts. They felt this would empower people to make more informed choices about both starting and stopping antidepressants.

I also think probably social media is the way to go, and also maybe people who are willing to say that they are a long-term antidepressant user and tell their story and their thoughts Just putting it out there would be fantastic there needs to be more awareness about the long-term effects of antidepressants on you and when is a good time and when there isn’t a good time to stop I think it would give people the platform to start reviewing their own, taking responsibility for stopping. (P16, F, 60-70yrs, 10+yrs on antidepressants)

## Discussion

31 RELEASE trial intervention arm participants with an average duration of antidepressant use of 16.5 years (range 1–30 years) were interviewed. Exposure to the RELEASE resources had prompted all to reconsider their antidepressant use, and 20 to start decreasing their antidepressant dose, eight of whom managed to successfully stop antidepressants by the time of the interview. Across seven themes, participants described how the RELEASE toolkit resources prompted reflection on their antidepressant use, supported informed conversations with GPs, and offered tapering plans that were both structured enough to instil confidence and flexible enough to help participants feel in control of their tapering journey. They also identified barriers, including beliefs that very small tapering doses were unnecessary, difficulty accessing the mini-doses required for hyperbolic tapering, and the challenge of managing withdrawal symptoms. For those who successfully stopped, the transition was associated with a renewed sense of autonomy. Participants also offered practical suggestions to strengthen the toolkit and its broader implementation.

### Implications for implementation and future research

The value of an intervention that engages both GPs and patients was evident in the findings. GPs can influence participants to either stay on or attempt to stop their antidepressant, playing a critical role in initiating tapering discussions and guiding patients through the process, a finding that is consistent with previous research.^47^,^50^ Providing patients with the RELEASE toolkit resources enabled and empowered patients to also initiate these conversations. This has important implications for broader implementation of the RELEASE toolkit in practice, suggesting that engaging GPs and patients should remain central. Likewise, participants described how the RELEASE resources encouraged them to reconsider their antidepressant use, highlighting the value of incorporating awareness-raising elements that prompt medication review and GP engagement.

Drug-specific structured tapering plans can empower long-term users by helping patients to visualise a way forward, providing the confidence to try. Flexibility enabled patients to remain in control of their own tapering journey. Nevertheless, many participants did not follow the tapering plans through the lower doses, sometimes believing that hyperbolic tapering to such small doses was not necessary or that accessing the requisite mini-doses was too difficult or expensive. Only two participants prepared their own liquid mini-doses as a low-cost solution, with many reporting this option as too complex, too time-consuming and/or too likely to result in imprecise dosing. Easy access to affordable mini-doses required for hyperbolic tapering represents a substantial barrier to implementation. In their absence, hyperbolic tapering will remain largely inaccessible for many people. This has important implications for future research and clinical practice. In the Netherlands, tapering-strips (ie, connected small plastic pouches), for hyperbolic reduction of antidepressant dose are available.^29 51^

Our findings confirm that withdrawal symptoms often disrupted participants’ tapering progress, consistent with findings from earlier research.^52^,^55^ These symptoms were often distressing and led some participants to give up on tapering altogether. While the RELEASE toolkit includes a resource for family and friends to support stopping antidepressants, some participants reported feeling pressured by family members to stay on or restart medication, which at times undermined their confidence and complicated their efforts to discontinue treatment.

Successfully stopping antidepressants fostered a sense of liberation and confidence. Success appeared to be more common in those with a shorter duration of antidepressant use (5.6 years vs 19.8 years for those who started tapering but resumed antidepressant), reinforcing that deprescribing conversations earlier rather than later are critical. Studies have shown that people who had been on antidepressants for a longer time were more likely to experience severe, long-lasting withdrawal symptoms and were less successful in stopping.^55^,^57^

The findings highlight the need for a wider range of supportive resources to facilitate successful antidepressant tapering. Participants recommended modifications that could enhance broader implementation such as apps to track tapering progress and withdrawal symptoms, as well as websites, telephone support lines and face-to-face psychological support services during tapering. To strengthen any broader implementation, future research should examine the feasibility and acceptability of apps, and identify which individuals are most likely to benefit from additional support. Participants also emphasised the importance of improving public awareness of overuse of antidepressants, withdrawal symptoms and tapering, underscoring the need for research on effective population-level awareness-raising strategies.

### Strengths and limitations

There are several strengths to the study. The reflexive thematic analysis was supported by regular analytic discussions where team members questioned each other’s ideas to make sure interpretations genuinely reflected the data. Variation in participants’ age, duration of antidepressant use, type of antidepressant used and stage of tapering is also a strength, providing a wide range of experiences and identification of shared and contrasting experiences. Few participants followed the final steps of the hyperbolic tapering, meaning the findings may not adequately capture the experience of mini-dose reductions required for hyperbolic tapering. Interview participants were recruited from within a trial setting, so findings may not fully represent the experiences of tapering outside this setting. People who agreed to be interviewed may hold stronger interest in engaging with the RELEASE toolkit resources than people who chose not to participate, meaning some perspectives may not be represented. Also, all 31 participants indicated at least an intention to stop antidepressants at some time in the future, this may have been part of their motivation to take part in the interview, so the findings do not capture the experiences of those who did not want to stop antidepressants. Despite this, the findings are informative for real-world implementation.

## Conclusions

Conducting the qualitative interviews within an effectiveness-implementation hybrid type-1 trial enabled timely insights into how the RELEASE toolkit was experienced, highlighting both what worked well and where refinements are needed. Findings suggest that the RELEASE toolkit resources played a meaningful role in prompting and supporting informed shared decision-making and guiding participants’ attempts to stop antidepressants. The structured nature of the tapering plans was valued giving participants the confidence to try to stop their antidepressant, while the flexibility enabled them to remain in control of their tapering journey. Considerations for modifications include clearer messaging about the potential severity of withdrawal symptoms, especially at lower antidepressant doses. Access to affordable mini-doses is a key system level barrier. Some participant suggestions have already been implemented, including making the RELEASE toolkit resources widely and freely available (releasetoolkit.com.au).^58^

## Data Availability

Data are available on reasonable request.
