# Supplementary material for: Toolkit to prompt and support stopping antidepressants in general practice: an interview study exploring patient participants’ experiences in the RELEASE trial
Source: BMJ Open. 2026 May 21;16(5):e116702. doi: 10.1136/bmjopen-2026-116702 (PMC13201990; doi:10.1136/bmjopen-2026-116702)
Supplement: Supplementary file 1 [file bmjopen-2026-116702.reviewer_comments.pdf]

PEER REVIEW HISTORY

BMJ Open publishes all reviews undertaken for accepted manuscripts. Reviewers are asked to complete a checklist review form and are provided with free text boxes to elaborate on their assessment. These free text comments are reproduced below.

ARTICLE DETAILS

Title (Provisional)

A toolkit to prompt and support stopping antidepressants in general practice: An interview study exploring patient participants' experiences in the RELEASE trial

Authors

MOURA FERREIRA, Polyana; McDonald, Suzanne; Wallis, Katharine A; Donald , Maria

---

VERSION 1 - REVIEW

**Reviewer**	**1**
**Name**	**Hølmkjær, Pernille**
**Affiliation**	**University of Copenhagen**
**Date**	**23-Feb-2026**
**COI**	**None**

Thank you for the opportunity to review your manuscript with an important focus on tampering antidepressants. I highly value the effort put into getting the patients perspective on the trial as well as the effectiveness of the intervention covered in the cRCT.

However, to better understand the focus of the manuscript and getting the results more clearly out, I have some suggestions and major revisions.

Concerning the review checklist:

2) Abstract:

I would suggest moving the explanation of the trial to the "study design" and keeping the "objective" clean with only the text concerning the objective for the study.

For "study design" I would also recommend to explicitly write "individual, semi-structured interviews".

Also, since there are 7 themes, it's a bit complicated to fully comprehend what the results entail. If possible, try to cluster them and do a more joint summary. Also see further suggestions for the result section.

4) Method:

It is written that the interview guide used open-ended questions and was adapted throughout the process of doing the interviews. However, since there is no interview guide as an appendix, it is not possible to know how much has been adapted and what the adaptations constituted.

10) Results:

The results section could benefit from a rewrite. 7 themes are presented, but when reading the themes, there is a bit of redundance and a lot of quotes which although they support the findings, make the readability somewhat difficult since there are so many. I would suggest trying to remove some of the quotes (could be presented in an appendix with a reference if needed), and maybe condense some of the text to improve readability.

Overall comments:

1) Overall, I'm a bit unsure if this is a genuine evaluation/process evaluation of the main trial or if it's an attempt to capture views on tampering antidepressants in general. Reading the main text, I feel inclined to see it as an evaluation, but I do believe it should be spelled out more. One suggestion could be to change the title of the manuscript to include RELEASE, this way it becomes more transparent, that the manuscript is a qualitative evaluation of the RELEASE intervention.

2) Method:

a. Please elaborate a bit on the study design. Is it a qualitative evaluation? I would suggest moving the "data was collected" comment to the actual part of "data collection" to avoid to much redundance in text.

b. Please include in the method selection when during the intervention period the interviews took place.

c. Please include a bit more details about the two people doing the interviews. Are they part of the original trial? Did they have any interest in seeing the RELEASE toolkit succeed? What were their backgrounds?

d. The number of participants should not be mentioned in the method section but kept in the results. I would suggest the sentence concerning how many were recruited from each GP office to be moved to the results section.

e. The "data analysis" part is very detailed and greatly written.

3) Results:

a. Is it correctly understood that all 31 participants wanted to stop AD? You did not interview anyone who did not want to stop? Maybe elaborate a bit on this in the limitation?

b. Overall, the results section is a bit long and with a lot of quotes. It makes it a bit difficult to read. Furthermore, when reading the themes, I feel it's possible to combine themes 1 and 2, and combine themes 3 and 4 and shorten some of the text.

For themes 1 and 2, these two themes describe the informed decision-making, covering the actual RELEASE-material and the GPs role. Maybe combining in one theme and then subthemes could sharpen the text and add a bit more clarity to the whole results section.

For themes 3 and 4, these two themes describe the actual tapering plans - with pros and cons. However, when split into 2 themes and subthemes, I feel there is a lot of redundance.

Based on the start of the discussion, I believe the manuscript would benefit from a more direct framing on the RELEASE-trials results from a qualitative viewpoint, which would also be more in line with the start of the discussion and the conclusion. It is mainly the title and the study design which would need some clarification. Then in the discussion, it could be further discussed how this evaluation can add to the overall knowledge on tampering.

Limitations:

Could you elaborate on what effect it had that the interviews took place during the intervention period? Could you imagine it could enhance/worsen the findings?

**Reviewer**	**2**
**Name**	**Johnson, Chris**
**Affiliation**	**NHS GGC, Pharmacy Services**
**Date**	**10-Mar-2026**
**COI**	**None**

Thank you for the opportunity to review the manuscript titled: *A toolkit to support stopping antidepressants in general practice: An interview study exploring patient experiences.*

This qualitative study explores a relative topic, antidepressant withdrawal, however participants experiences were limited to hyperbolic reduction, and non-hyperbolic reduction were not assessed. Nor does the introduction discuss previous studies assessing withdrawal.

Main issues:

- Strengths and limitations, after abstract – add bullet point. *Lack of perspectives on non-hyperbolic reduction*
- Methods section requires reorganisation to aid clarity. Study not reported inline with qualitative reporting guidance.
- Sampling method, invite to participate and consent needs to be more clearly stated.
- Conclusion in main text appears presumptuous - *To enable broader implementation of the RELEASE toolkit in general practice* … and *the importance of hyperbolic dose tapering…* Contradicts participant experiences reported as some people stopped the hyperbolic reduction as they felt it was not needed, high cost and a faff.

Positives:

This paper highlights some of the challenges: lack of proactive medication reviews, lack of information regarding treatment duration, financial costs of hyperbolic reduction, and lack of need for hyperbolic reduction for all.

Introduction

Long-term antidepressant use, please acknowledge other potential drivers of long-term antidepressant use eg diabetic related neuropathic pain. Lack of proactive reviews by prescribers, the financial cost of seeing your GP in Australia and New Zealand being a barrier to reviewing and reducing when appropriate. The fear that prescribers have in reducing psychotropic medication (Svensson SA, et al. BMC Fam Pract 2019;20(1):4. Johnson CF, et al. BMC Fam Pract 2017;18(1):72 Maund E, et al. J Affective Disorders 2019;245:38-62)

Missing Lack of studies assessing different reduction methods hyperbolic v non-hyperbolic.

Please add paragraph of literature/studies focusing on antidepressant approaches eg Maund E, Stuart B, Moore M, et al. Managing Antidepressant Discontinuation: A Systematic Review. Annals of Family Medicine 2019;17(1):52-60. Kendrick T, Stuart B, Bowers H, et al. Internet and Telephone Support for Discontinuing Long-Term Antidepressants: The REDUCE Cluster Randomized Trial. JAMA Network Open 2024;7(6):e2418383-e83. doi: 10.1001/jamanetworkopen.2024.18383

RELEASE study – please consider referencing study protocol, nearer start of paragraph 4, eg in line 50.

Line 11 therapeutic guidelines reference. Link unclear what in the Therapeutic Guideline Ltd is the paper refers to. Also consider adding guidelines which are relevant to internation readers eg NICE, American Psychiatric Association, British Association of Psychopharmacology.

Line 14 *'People failing to stop'.* Consider tempering language. Please consider, is it people failing to stop, or prescribers failing to proactively review people and create opportunity for appropriate reduction.

Line 36 only need to reference Horowitz ref 19 once. As other Horowitz refs 19 and 22 are just duplication of ref 19 (Lancet Psych paper).

Method

Page 5 – hyperbolic reduction – consider add Horowitz ref 19 to line 10.

Study design. Please move to start of section. Please expand. One to one interviews involving which qualitative analytical approach? Study does not appear to be reported in line with qualitative study reporting guidelines – please add.

Participant recruitment. Sampling strategy is too vague. Was purposive sampling used to get a breath and range of perspectives? Consider providing some demographic information on RELEASE study population in the trial section will help with context with the qualitative study population e.g. RELEASE median age was 40 (range 26-76)

Data collection

Interview guide – was there PPI involvement in its design/content? What informed the guide content? Needs to be stated clearly.

When was participant consent obtained, and how was that obtained? Any follow up email after first email to participate?

Ethical approval hidden. Please move to more visible area eg last sentence of study design section.

Data analysis

RTA??? Please write in full. Used 5 times and more commonly associated with Road Traffic Accidents which may subconsciously influence readers perspectives of this study. Add Ref for RTA page 7 line 7 end of first sentence.

Word count and repetition - RELEASE study descriptions consider moving all Intro. Having in one section would reduce duplication and save lots of words…. Possibly consider a panel/figure to outline main RELEASE components.

PPI section – consider include where interview schedule is first discussed.

Results

Sample size rational – does not belong in results section

Page 7 – please restructure to improve clarity.

How did the venlafaxine/desvenlafaxine participants experiences compare to others? As venlafaxine/desvenlafaxine is known to be problematic.

Quotations. Please consider minimising the length of descriptor, such as reducing to P9, F 51y. As the paper has already described the population in Table 1, and explanatory text prior to explanatory quotes is clear. The long descriptors, from a readers point of view, are creating unhelpful noise that is detracting from the power of the quote(s).

Data from participants previous experience/perspectives on stopping should be considered and included – what difference did RELEASE provide?

Discussion

Page 19 lines 49 to 60. Please consider summarising your findings, to get to the point. Maybe ‘Long-term antidepressant use is a growing challenge, therefore enabling review and appropriate reductions in prescribing is important in enabling recovery. The RELEASE toolkit…. to stop antidepressants. This study identified seven key themes….’

Clear Comparison with literature section missing

Strengths and limitation section very brief. Lots of potential limitations to consider discuss e.g. no non-hyperbolic reduction perspectives, or comments from participants, although some quotes seem to hint at pros and cons with different approaches.

Implications for research and practice could be more clearly stated – eg a clear section.

Conclusion

Please moderate

'To enable broader implementation of the RELEASE toolkit in general practice…' Appears to be presumptuous what is the outcome of the RELEASE trial. And see comment above.

Tapering plan easy access. This resource may be of help and provides a range of non-hyperbolic and hyperbolic reduction options:

- Quality Prescribing for Antidepressants: A Guide for Improvement 2024-27
- App version of above: Antidepressants: Quality prescribing - a guide for improvement | Right Decisions
    - Reduction schedules: Reducing and stopping antidepressants | Right Decisions

---

VERSION 1 - AUTHOR RESPONSE

**Reviewer 1**	**Response**
Abstract: I would suggest moving the explanation of the trial to the "study design" and keeping the "objective" clean with only the text concerning the objective for the study.	Thank you for this suggestion, however we feel it would be confusing for the reader to present the details of the trial in the study design section of this manuscript, which reports a qualitative research study.
Abstract: For "study design" I would also recommend to explicitly write "individual, semi-structured interviews".	We agree and the study design now states: "One-to-one semi-structured qualitative interviews using reflexive thematic analysis to identify patterns of shared meaning."
Abstract: Also, since there are 7 themes, it's a bit complicated to fully comprehend what the results entail. If possible, try to cluster them and do a more joint summary. Also see further suggestions for the result section.	Thank you for this comment. We have provided a simplified summary of the 7 themes in the abstract as well as in the first paragraph of the Discussion.
Method: It is written that the interview guide used open-ended questions and was adapted throughout the process of doing the interviews. However, since there is no interview guide as an appendix, it is not possible to know how much has been adapted and what the adaptations constituted.	Only a minor adaptation was made, which we have now clarified in the text in the data collection section of the methods.
Results: The results section could benefit from a rewrite. 7 themes are presented, but when reading the themes, there is a bit of redundance and a lot of quotes	Thank you for this suggestion. We have removed eight quotes to improve readability and revised some of the text in the results.

which although they support the findings, make the readability somewhat difficult since there are so many. I would suggest trying to remove some of the quotes (could be presented in an appendix with a reference if needed), and maybe condense some of the text to improve readability.	
Overall comments: Overall, I'm a bit unsure if this is a genuine evaluation/process evaluation of the main trial or if it's an attempt to capture views on tampering antidepressants in general. Reading the main text, I feel inclined to see it as an evaluation, but I do believe it should be spelled out more. One suggestion could be to change the title of the manuscript to include RELEASE, this way it becomes more transparent, that the manuscript is a qualitative evaluation of the RELEASE intervention	We have clarified that the study is a qualitative evaluation of the RELEASE trial in both the manuscript title and study design section of the methods.
Method: a. Please elaborate a bit on the study design. Is it a qualitative evaluation? I would suggest moving the "data was collected" comment to the actual part of "data collection" to avoid to much redundance in text.	Thank you for this suggestion. In addition to clarifying that this is a qualitative evaluation, we have moved the "data was collected" comment to the data collection section. We have also separated the "participant recruitment" and "data collection" sections to improve clarity.
Method: b. Please include in the method selection when during the intervention period the interviews took place	We have added a sentence to the data collection section to clarify that interviews were conducted during the 12-month follow-up period of the trial. Table 1 also provides the mean time between allocation to the intervention arm of the trial and the interview (mean was 10.5 months).
Method: c. Please include a bit more details about the two people doing the interviews. Are they part of the original trial? Did they have any interest in seeing the RELEASE toolkit succeed? What were their backgrounds?	Thank you for pointing that out, we have added a phrase to the text to clarify that the two interviewers were not involved in delivering the intervention. The authors' backgrounds and perspectives are described in the reflexivity paragraph under the data analysis section.
Method: d. The number of participants should not be mentioned in the method section but kept in the results. I would	In line with a request from the Editor, we report the number of participants interviewed in the Methods section as well as in the results.

suggest the sentence concerning how many were recruited from each GP office to be moved to the results section.	
Method e. The “data analysis” part is very detailed and greatly written.	Thank you. All your comments are really appreciated.
Results: a. Is it correctly understood that all 31 participants wanted to stop AD? You did not interview anyone who did not want to stop? Maybe elaborate a bit on this in the limitation?	Yes, all 31 expressed at least an intention to stop antidepressants at some time in the future, so we agree this is a limitation and have added a sentence to the limitations section of the discussion.
Overall, the results section is a bit long and with a lot of quotes. It makes it a bit difficult to read. Furthermore, when reading the themes, I feel it's possible to combine themes 1 and 2, and combine themes 3 and 4 and shorten some of the text.<br>For themes 1 and 2, these two themes describe the informed decision-making, covering the actual RELEASE-material and the GPs role. Maybe combining in one theme and then subthemes could sharpen the text and add a bit more clarity to the whole results section.<br>For themes 3 and 4, these two themes describe the actual tapering plans - with pros and cons. However, when split into 2 themes and subthemes, I feel there is a lot of redundance.	The themes were carefully crafted by the research team across several analytical team meetings, using the reflexive thematic approach. We believe all seven themes are necessary to accurately represent the breadth of participant experiences and to maintain the integrity of the analysis. To support readability, we have however reduced the number of illustrative quotes and modified some text.
Based on the start of the discussion, I believe the manuscript would benefit from a more direct framing on the RELEASE-trials results from a qualitative viewpoint, which would also be more in line with the start of the discussion and the conclusion. It is mainly the title and the study design which would need some clarification. Then in the discussion, it could be further discussed how this evaluation can add to the overall knowledge on tampering.	We agree and we have clarified in the title and the study design that the study is a qualitative evaluation of the RELEASE trial from the patients' perspective.
Limitations: Could you elaborate on what effect it had that the interviews took place during the intervention period?	Hybrid type-1 designs aim to concurrently investigate effectiveness and gather data on context, which can help identify timely insights to accelerate translation into practice once

Could you imagine it could enhance/worsen the findings?	effectiveness is known. Consistent with this design, the evaluation interviews were conducted alongside the trial. Any potential influence of the interviews on participant experiences or outcomes is inherent to this study design. We have added text in several places throughout the manuscript to highlight that the interviews were part of the implementation evaluation for the effectiveness-implementation hybrid type-1 cluster randomised controlled trial.

**Reviewer: 2**	**Response**
Main issues:<br>• Strengths and limitations, after abstract – add bullet point. Lack of perspectives on nonhyperbolic reduction	We understand the interest in non-hyperbolic reduction; however, it was not a requirement that participants followed a hyperbolic method of tapering, and indeed the interviews demonstrate that few participants did follow this method of tapering. But for clarity we have reworded the third bullet point in the strengths and limitations.
• Methods section requires reorganisation to aid clarity. Study not reported inline with qualitative reporting guidance.	Methods section has been reworded and separated into two sections, one for participant recruitment and one for data collection, to increase clarity.<br>This data was analysed using Braun and Clarke's reflexive thematic analysis who recommend against rigidly applying reporting checklists such as the COREQ, nevertheless we have completed the checklist to satisfy editorial and reviewer requests (Supplementary File 1). See Braun, V., & Clarke, V. (2021). One size fits all? What counts as quality practice in (reflexive) thematic analysis? Qualitative Research in Psychology, 18(3), 328–352.
• Sampling method, invite to participate and consent needs to be more clearly stated.	Thank you for this suggestion, we have rewritten the participant recruitment section to improve clarity.
• Conclusion in main text appears presumptuous - To enable broader implementation of the RELEASE toolkit in general practice … and the importance of hyperbolic dose tapering… Contradicts participant experiences reported as some	Thank you for pointing this out, we agree and have removed the statement from the Conclusion. The Conclusion has been revised to emphasise that the qualitative interviews were part of an effectiveness-implementation hybrid type-1 cluster randomised controlled

people stopped the hyperbolic reduction as they felt it was not needed, high cost and a faff.	trial highlighting both what worked well and where refinements are needed.
Positives: This paper highlights some of the challenges: lack of proactive medication reviews, lack of information regarding treatment duration, financial costs of hyperbolic reduction, and lack of need for hyperbolic reduction for all.	Thank you. We appreciate all suggestions.
Introduction: Long-term antidepressant use, please acknowledge other potential drivers of long-term antidepressant use eg diabetic related neuropathic pain. Lack of proactive reviews by prescribers, the financial cost of seeing your GP in Australia and New Zealand being a barrier to reviewing and reducing when appropriate. The fear that prescribers have in reducing psychotropic medication<br>(Svensson SA, et al. BMC Fam Pract 2019;20(1):4.<br>Johnson CF, et al. BMC Fam Pract 2017;18(1):72<br>Maund E, et al. J Affective Disorders 2019;245:38-62)	Thank you for this suggestion, we have included other potential drivers of long-term antidepressant use in the Introduction, including citations as suggested.
Introduction: Missing Lack of studies assessing different reduction methods hyperbolic v non-hyperbolic.	The purpose of this work was to explore patient participants' experiences of antidepressant reduction within the context of the RELEASE trial, rather than to evaluate the relative effectiveness of different tapering strategies. For this reason, we believe that including a comparison of hyperbolic versus non-hyperbolic approaches in the introduction is outside the scope of the manuscript.
Introduction: Please add paragraph of literature/studies focusing on antidepressant approaches eg:<br>Maund E, Stuart B, Moore M, et al. Managing Antidepressant Discontinuation: A Systematic Review. Annals of Family Medicine 2019;17(1):52-60.<br>Kendrick T, Stuart B, Bowers H, et al. Internet and Telephone Support for Discontinuing Long-Term	A summary of studies investigating antidepressant approaches including relevant citations has been added to the Introduction.

Antidepressants: The REDUCE Cluster Randomized Trial. JAMA Network Open 2024;7(6):e2418383-e83. doi:10.1001/jamanetworkopen.2024.18383	
Introduction: RELEASE study – please consider referencing study protocol, nearer start of paragraph 4, eg in line 50	We have added the reference for the study protocol.
Introduction: Line 11 therapeutic guidelines reference. Link unclear what in the Therapeutic Guideline Ltd is the paper refers to. Also consider adding guidelines which are relevant to internation readers eg NICE, American Psychiatric Association, British Association of Psychopharmacology.	Thank you, we have removed the Australian Therapeutic Guidelines reference as it requires a subscription to access and have added the NICE guidelines and the American Psychiatric Association citations.
Introduction: Line 14 'People failing to stop'. Consider tempering language. Please consider, is it people failing to stop, or prescribers failing to proactively review people and create opportunity for appropriate reduction.	We agree and have reworded.
Introduction: Line 36 only need to reference Horowitz ref 19 once. As other Horowitz refs 19 and 22 are just duplication of ref 19 (Lancet Psych paper).	References removed.
Method: Page 5 – hyperbolic reduction – consider add Horowitz ref 19 to line 10	Reference added.
Study design. Please move to start of section. Please expand. One to one interviews involving which qualitative analytical approach?	Moved and rephrased.
Study does not appear to be reported in line with qualitative study reporting guidelines – please add	Please see earlier response. This data was analysed using Braun and Clarke's reflexive thematic analysis who recommend against rigidly applying reporting checklists such as the COREQ, nevertheless we have completed the checklist to satisfy editorial and reviewer requests (Supplementary File 1).See Braun, V., & Clarke, V. (2021). One size fits all? What counts as quality practice in (reflexive) thematic analysis? Qualitative Research in Psychology, 18(3), 328–352.

Participant recruitment. Sampling strategy is too vague. Was purposive sampling used to get a breath and range of perspectives?	We have clarified the sampling strategy in the "Participant recruitment" section
Consider providing some demographic information on RELEASE study population in the trial section will help with context with the qualitative study population e.g. RELEASE median age was 40 (range 26-76).	We understand how this would be helpful for context, but as the main RELEASE trial analyses are still ongoing and will be reported separately, we are unable to include trial demographic data at this stage.
Data collection: Interview guide – was there PPI involvement in its design/content? What informed the guide content? Needs to be stated clearly	We have reworded to clarify the LEAGs involvement. We have added a sentence to the "Data collection" section about how the guide was developed.
When was participant consent obtained, and how was that obtained? Any follow up email after first email to participate?	We have rewritten the "Participant recruitment" section to clarify how and when consent was obtained. Up to two reminders were sent, and we have specified this in the "Participant recruitment" section.
Ethical approval hidden. Please move to more visible area eg last sentence of study design section.	We have added a heading called "Ethical Approval" to improve visibility.
Data analysis: RTA??? Please write in full. Used 5 times and more commonly associated with Road Traffic Accidents which may subconsciously influence readers perspectives of this study.	RTA is now written in full as reflexive thematic analysis.
Add Ref for RTA page 7 line 7 end of first sentence.	Reference added.
Word count and repetition - RELEASE study descriptions consider moving all Intro. Having in one section would reduce duplication and save lots of words…. Possibly consider a panel/figure to outline main RELEASE components.	Thank you for this suggestion, we have refined and moved the description of the RELEASE intervention and trial to the introduction.
PPI section – consider include where interview schedule is first discussed.	We have clarified that the Lived Experience Advisory Group functioned as an insight and advisory group, informing the broader focus of the study and did not have direct input into the interview schedule, so our preference is to keep the PPI section separate.

Results: Sample size rational – does not belong in results section	We agree and we have moved the sample size rational to the methods section.
Page 7 – please restructure to improve clarity.	Page 7 describes the findings presented in Table 1.
How did the venlafaxine/desvenlafaxine participants experiences compare to others? As venlafaxine/desvenlafaxine is known to be problematic.	We acknowledge that venlafaxine/desvenlafaxine can be more challenging to stop. However, exploring differences between medication types was not an aim of this qualitative study, which focused on participants' experiences of using the RELEASE intervention.
Quotations. Please consider minimising the length of descriptor, such as reducing to P9, F 51y. As the paper has already described the population in Table 1, and explanatory text prior to explanatory quotes is clear. The long descriptors, from a readers point of view, are creating unhelpful noise that is detracting from the power of the quote(s).	We agree and have minimised the length of the descriptor.
Data from participants' previous experience/perspectives on stopping should be considered and included – what difference did RELEASE provide?	Thank you for this suggestion. Unfortunately, we do not have comprehensive interview data on participants' prior experiences or perspectives on stopping. Where participants did mention previous stopping attempts, we have incorporated those insights when they were informative.
Discussion: Page 19 lines 49 to 60. Please consider summarising your findings, to get to the point. Maybe 'Long-term antidepressant use is a growing challenge, therefore enabling review and appropriate reductions in prescribing is important in enabling recovery. The RELEASE toolkit…. to stop antidepressants. This study identified seven key themes….'	Thank you for this helpful observation. We have summarised the seven themes in the first paragraph.
Clear Comparison with literature section missing	Comparison with relevant literature is integrated throughout the Discussion. Paragraphs 2 through 5 explicitly draw on and engage with prior research on antidepressant discontinuation, digital support tools, and patient experiences of tapering. In addition, the journal guidelines recommend a Discussion of no more than five paragraphs, we feel the current discussion adequately

	addresses the relevant issues within the required length.
Strengths and limitation section very brief. Lots of potential limitations to consider discuss e.g. no non-hyperbolic reduction perspectives, or comments from participants, although some quotes seem to hint at pros and cons with different approaches.	We have added additional limitations to the Discussion section and to the bullet point section after the abstract. The following has been added to the Discussion: "People who agreed to be interviewed may hold stronger interest in engaging with the RELEASE toolkit resources than people who chose not to participate, meaning some perspectives may not be represented. Also, all 31 participants indicated at least an intention to stop antidepressants at some time in the future, this may have been part of their motivation to take part in the interview, so the findings do not capture the experiences of those who did not want to stop antidepressants."<br><br>The interviews highlight that few participants followed the hyperbolic tapering method (see for example the sub-theme: perceived lack of need for hyperbolic tapering through to very low doses) so we don't believe that having 'no non-hyperbolic reduction perspectives' is a limitation.
Implications for research and practice could be more clearly stated – eg a clear section.	We have added a heading "Implications for implementation and future research" to make this section clearer.
Conclusion: Please moderate 'To enable broader implementation of the RELEASE toolkit in general practice…' Appears to be presumptuous what is the outcome of the RELEASE trial. And see comment above.	We agree and have revised the text in the Conclusion accordingly.
Tapering plan easy access. This resource may be of help and provides a range of nonhyperbolic and hyperbolic reduction options:<br><br>• Quality Prescribing for Antidepressants: A Guide for Improvement 2024-27<br><br>• App version of above: Antidepressants: Quality prescribing - a guide for improvement | Right Decisions<br><br>Reduction schedules: Reducing and stopping antidepressants | Right	Thank you for highlighting these resources.

VERSION 2 - REVIEW

**Reviewer**	**1**
**Name**	**Hølmkjær, Pernille**
**Affiliation**	**University of Copenhagen**
**Date**	**16-Apr-2026**
**COI**	

Thank you for a thorough rewrite of the manuscript and addressing all my concerns and questions.

Although I still believe there is some redundance in the results when having 7 themes, I fully acknowledge that I do not have access to the raw material (and it is 31 interview - it's a lot!) and that you have a done a great job with how the analysis have been made.

It was merely a suggestion since I thought it could have given a bit more, but there is nothing wrong with how it is, and the removal of some of the quotes have definitely improved the readability.

I have no further inquiries or suggestions - good luck.

**Reviewer**	**2**
**Name**	**Johnson, Chris**
**Affiliation**	**NHS GGC, Pharmacy Services**
**Date**	**15-Apr-2026**
**COI**	

Thank you for address all comments, and tracking these clearly.
